# Supplementary material for: Current-induced skyrmion generation and dynamics in symmetric bilayers
Source: Nat Commun. 2017 Jun 8;8:15765. doi: 10.1038/ncomms15765 (PMC5472776; doi:10.1038/ncomms15765)
Supplement: Supplementary Information — Supplementary Figures, Supplementary Table, Supplementary Notes and Supplementary References [file ncomms15765-s1.pdf]

Supplementary Note 1. SAMPLE CHARACTERIZATION

1. Magnetometry measurements

The sample symmetry is a key point in this work. In order to check our ability to grow the magnetic films with similar parameters for both Pt\FM\Au and Au\FM\Pt stack order, we have characterized samples containing only one magnetic layer. All films were grown on a Ta(3 nm)\Pt(5 nm) buffer layer deposited on Si(001) with a native oxide layer. For the Pt\FM\Au sample, the FM [Ni(4 Å)\Co(7 Å)\Ni(4 Å)] layer has been grown directly on the buffer layer, while for the Au\FM\Pt, a 5 nm thick Au layer was grown before the FM. The cover layer (Au and Pt respectively) was 5 nm thick. These samples were compared to the magnetic bilayer sample Pt\FM\Au(d)\FM\Pt with $d = 3$ nm and $d = 5$ nm.

Using SQUID, the saturation magnetization $M_s = 0.85 \times 10^6$ A m^{-1} has been determined on all single and bilayer samples, and is close to the value (0.9×10^6 A m^{-1}) expected from the bulk magnetization of Co (1.37×10^6 A m^{-1}) and Ni (0.49×10^6 A m^{-1}).

Supplementary Fig. 1a shows hysteresis loops determined using magneto-optical polar Kerr effect, as a function of an out-of-plane magnetic field for both single layer samples and two bilayer samples which differ by the spacer thickness d. While both single layers show similar square hysteresis, i.e. full remanence and similar coercive field, the bilayer samples have a lower remanence indicating spontaneous tendency to demagnetization. Note that the thicker the spacer, the smaller the saturation field, which indicates a decreased coupling.

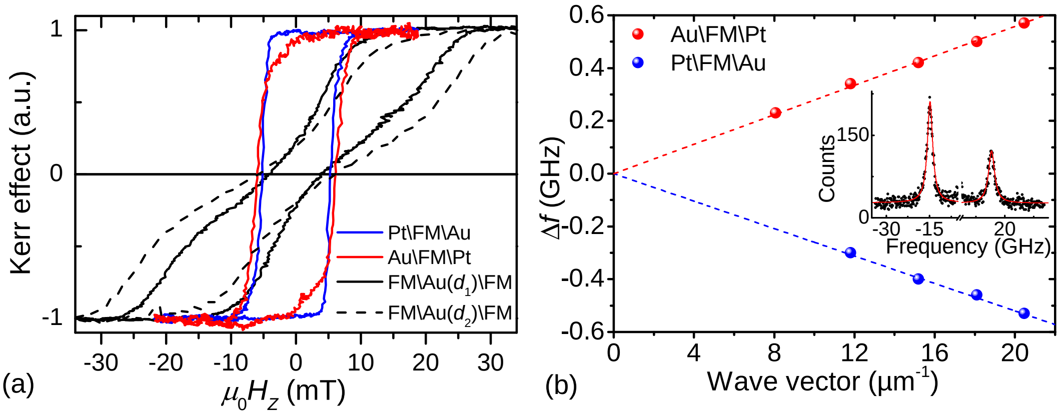

Supplementary Figure 1 | Stack characterization. (**a**) Hysteresis loops of the single magnetic layer of Pt/FM/Au and Au/FM/Pt where FM=Ni(4 Å)\Co(7 Å)\Ni(4 Å), and of corresponding magnetic bilayer with different thickness of Au spacer $d_1 = 5$ nm and $d_2 = 3$ nm. (**b**) Frequency shift $\Delta f = f_S - f_{AS}$ obtained in Pt/FM/Au (blue) and Au/FM/Pt (red) stacks. The inset shows an example of BLS spectra measured at 20.45 μm^{-1} in Pt/FM/Au, under a 0.6 T in-plane field, with red lines corresponding to Lorentzian fits.

2. Brillouin light scattering

The DMI has been quantified by the Brillouin light scattering (BLS) method[1,2], in Damon-Eshbach geometry (in-plane magnetization, spin wave propagation vector perpendicular to the magnetization). Here two oppositely propagating spin waves defined by wave vector k_x are sensitive to the DMI parameter D causing a frequency shift Δf between Stokes (spin-wave creation) and anti-Stokes (spin-wave annihilation) modes. An example of Stokes and anti-Stokes peaks for an in-plane magnetic saturating field $\mu_0 H_y = 0.6$ T is displayed in Supplementary Fig. 1b.

To access the DMI in the individual layers, we have measured the $\Delta f(k_x)$ dependence on single Pt/FM/Au and Au/FM/Pt layers. The difference between Stokes and anti-Stokes frequencies $\Delta f = f_S - f_{AS} = 2\gamma k_x D/\pi M_s$, shown in Supplementary Fig. 1b allows extraction of the DMI parameter from the linear fits. We find effective values of $D = -0.21 \pm 0.01$ mJ m^{-2} for Pt/FM/Au and $D = +0.24 \pm 0.01$ mJ m^{-2} for Au/FM/Pt stacks, respectively.

Additionally, BLS spectra provide an estimation of the magnetic anisotropy and the damping factor. The resonance frequency is $f_r = \sqrt{f_x f_z}$, where the two principal frequencies read $f_x = (\gamma_0/2\pi)H_y$ and $f_z = (\gamma_0/2\pi)(H_y - H_K)$, where H_K is the effective anisotropy field. These two frequencies shift linearly in k_x in the presence of DMI, but the shift is small so we perform calculation at zero wave vector. From $\mu_0 H_y = 0.6$ T and the gyromagnetic factor $g = 2.17$ for Co, one estimates $f_x = 18.2$ GHz, hence from the experimental $f_r = 15.75$ GHz, defined as the mean between Stokes and anti-Stokes frequencies, one gets $f_z = 13.6$ GHz. The anisotropy field is then $\mu_0 H_K = 152$ mT. Subtracting the shape anisotropy $K_d = \frac{1}{2}\mu_0 M_s^2$, the sample anisotropy is 0.52 MJ m^{-3}. From the expression of the imaginary part of the magnetic susceptibility one gets, for small damping, that the full width at half-maximum (FWHM, in the frequency domain) δf of the BLS peak is $\delta f = \alpha(f_x + f_z)$. Assuming that no inhomogeneous broadening exists, the FWHM of the BLS peak thus provides an upper limit for the Gilbert damping α. On the spectrum shown in Supplementary Fig. 1b one measures $\delta f = 2.5 \pm 0.3$ GHz. From this we find $\alpha \leq 0.08 \pm 0.01$.

3. Interlayer exchange coupling

In order to verify that the electronic coupling in our bilayer is eliminated by the choice of 3 nm Au spacer, we have grown an unbalanced sample with stronger perpendicular anisotropies in order to obtain two square hysteresis loops with different coercive fields: Pt(5 nm)\Co(5 Å)\Ni(6 Å)\Au(3 nm)\Ni(5 Å)\Co(5 Å)\Ni(5 Å)\Pt(5 nm). The major and minor hysteresis loops are shown in Supplementary Fig. 2. The two observed steps correspond to magnetization reversal in the first and second layer. The electronic coupling between both layers can be deduced from the bias field in the minor loops. Since no bias field is observed, as shown from the perfect superposition between minor loops obtained with a hard layer saturated up or down, the interlayer exchange coupling is negligible for a 3 nm spacer, in agreement with previous investigation on Co/Au/Co system[3].

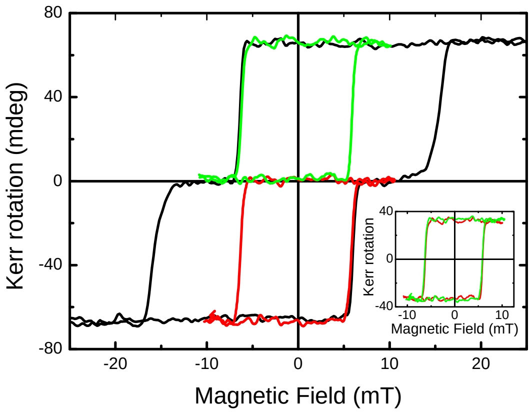

Supplementary Figure 2 | Interlayer coupling. Full and minor hysteresis loops measured on Pt(5 nm)\Co(5 Å)\Ni(6 Å)\Au(3 nm)\Ni(5 Å)\Co(5 Å)\Ni(5 Å)\Pt(5 nm) by polar Kerr magnetometery. The inset shows superimposition of the two minor loops.

Supplementary Note 2. STABILIZATION MECHANISM VIA DIPOLAR COUPLING

This paper proposes a new mechanism, based on dipolar couplings, for chiral DWs stabilization by lowering its energy, key towards skyrmion stabilization. While in the main text, and in particular in Fig. 1b, the demonstration is performed on an isolated DW, we extend here the discussion to the more complex situations of 360° DWs and skyrmions.

Dipolar couplings have been shown to lower the DW energy in the symmetric bilayer DWs, through a flux closure mechanism. This implies long range interaction of the stray field emitted by the domains with the DW magnetization. In a skyrmion situation, schematically, the strength of the interaction should reduce as the two walls on either side

of the skyrmion core come close to each other, which lowers the stray field coming out from the skyrmion core. Here we show that this picture based on the isolated DWs remains unchanged down to 40 nm separated DWs.

We first consider a 360° DW. In such a situation, two chiral 180° DWs are separated by a fixed distance d_0. The distance can be controlled by an external perpendicular magnetic field H_z, oriented against the magnetization of the domain situated between the two DWs, as[5] $d_0 \propto 1/\sqrt{H_z}$. The domain wall energy is extracted from the micromagnetic simulation, where from the micromagnetic energy we subtract the Zeeman energy and the dipolar interaction energy between the domains, which corresponds to the dipolar energy of abrupt domain walls (zero thickness) as shown by Kooy and Enz[6]. The energy is then divided by the domain wall surface, given by the magnetic sample thickness t and the length of the domain wall (twice the simulation width for 360° domain walls to account for the two 180° walls). We have calculated the DW energy in single and bilayers (for a 3 nm thick spacer), as a function of d_0, as plotted in Supplementary Fig. 3. For a large separation, we find the same DW energy as for isolated DWs. As d_0 decreases, we observe an increase of the DW energy below about 40 nm, for both mono and bilayer DWs. Such an increase cannot be attributed only to the decay of the dipolar coupling mechanism. Indeed, as the distance becomes comparable to the DW width, the DW profile adapts to the compression of the 360° DW, so that all the micromagnetic energies are expected to increase. In single layers, the energy increase is only due to this effect so that this calculation serves as a reference. The DW energy gain due to the flux closure mechanism corresponds to the difference between the single and bilayer calculation. For large d_0, we find ≈ 0.7 mJ m^{-2} energy gain in bilayers similar to the result in Fig. 1b. This value is rather constant down to 40 nm separation then drops down for smaller separation. For a 10 nm distance, i.e. smaller than the DW width, the strength of flux closure effect is still 50% of that at large separation.

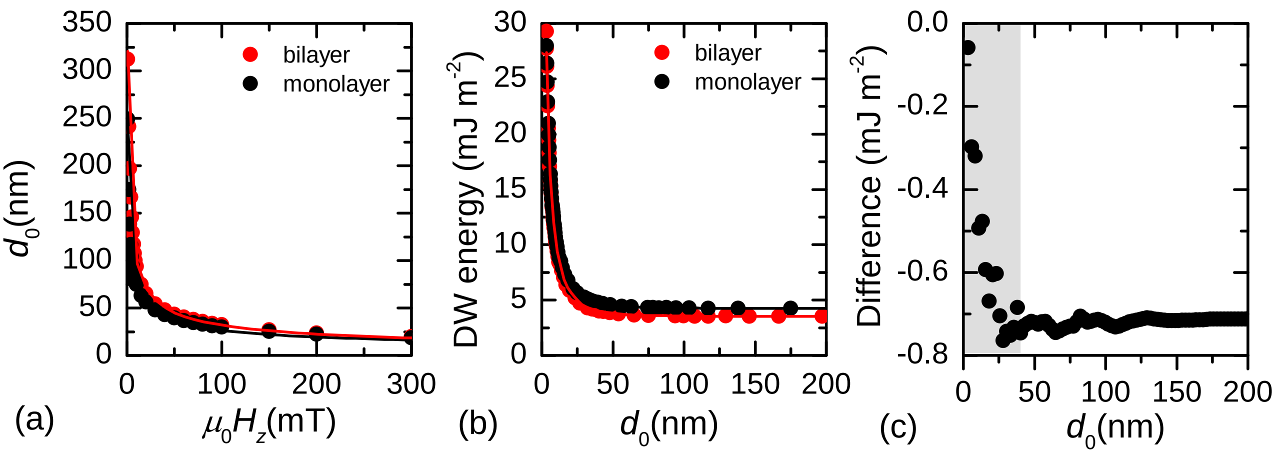

Supplementary Figure 3 | 360° DW stabilization (**a**) 360° DW separation d_0 as a function of the applied magnetic field for single and bilayer films. The dots correspond to the micromagnetic simulation and the lines to a $1/\sqrt{H_z}$ fit. (**b**) DW energy as a function of d_0. The full lines correspond to an interpolation used to calculate the energy difference. (**c**) DW energy difference between mono and bilayer films, which corresponds to the energy gain due to the flux closure mechanism. The low frequency variations are due to calculation and interpolation imprecisions and thus are meaningless. The decrease to zero below 40 nm (gray area) is due to the decay of the flux closure mechanism efficiency for small DW separations.

Performing the same calculation for skyrmions is more complicated, as skyrmions could not be stabilized in monolayer films and therefore could not be used as a reference. However, we have calculated the compression of skyrmions under a perpendicular field H_z oriented against the skyrmion core and extracted the DW energy. We find that, thanks to the external field, skyrmions can be compressed down to 20 nm diameter at 25 mT as shown in Supplementary Fig. 4a. The extracted DW energy (compared to the 360° domain wall calculation, here the dipolar interaction energy between the skyrmion core and the ferromagnetic surrounding is calculated from a uniform circular reversed domain of radius r_{s}, and the energy is divided by $2\pi r_{\mathrm{s}} t$, the domain wall surface in the skyrmion), displayed in Supplementary Fig. 4b, is found to increase as the skyrmion is compressed. At large skyrmion diameter, the DW energy is in good agreement with the isolated DW energy. The increase at small diameter can be attributed to the interaction between diametrically opposite DWs, similarly to what has been discussed in 360° DWs, as well as to the curvature of the DWs[7]. Using the curvature energy $\sigma_c \approx 2A\Delta/r_{\mathrm{s}}^2$ from Ref. 7 and the DW energy extracted from the 360° DW analysis, we reproduce quite well the DW energy. The difference can be attributed on one hand to the imprecision of the curvature energy formula (demonstrated for large skyrmions) and on the other hand to a further lowering of the flux closure mechanism presented in this study, as compared to 360° DWs. However, it turns out that,

whatever the decreased efficiency of the flux closure mechanism, such a mechanism still remains so that we are able to demonstrate, in the calculation, ultrasmall skyrmion stabilization. This opens perspectives for future experiments to stabilize smaller skyrmions. Tuning the micromagnetic parameters should allow ultrasmall skyrmions stabilization at smaller applied magnetic field than in this numerical study.

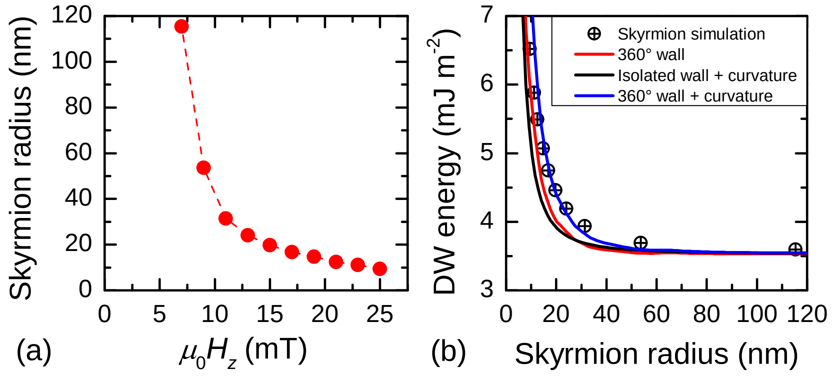

Supplementary Figure 4 | Skyrmion stabilization (a) Skyrmion radius variation as a function of the external field strength. At 6 mT, a good agreement is found with the experiment. (**b**) Extracted DW energy as a function of the skyrmion radius. The dotted line corresponds to the DW curvature energy, as calculated from Ref. 7, and the full line corresponds to the sum of the DW curvature energy and the DW energy as calculated form the 360° DW study.

Supplementary Note 3. SKYRMION DEFLECTION: COMPETITION BETWEEN GYROTROPIC AND OERSTED FIELD EFFECTS

The perpendicular magnetic field generated by electric current flowing into the stripe has a gradient in the transverse direction. Therefore it may cause a deflection of the skyrmion, and compete with the deflection caused by gyrotropic effects.

In our situation, the magnetic film is surrounded by conducting layers so that the Oersted field is essentially perpendicular to the stripe plane, and only depends on the transverse coordinate y:

$$B_z(y) = \frac{\mu_0 jh}{4\pi}\left[\ln\left(\frac{(h/2)^2+(w/2+y)^2}{(h/2)^2+(w/2-y)^2}\right)+8\frac{w/2+y}{h/2}\arctan\left(\frac{h/2}{w/2+y}\right)-8\frac{w/2-y}{h/2}\arctan\left(\frac{h/2}{w/2-y}\right)\right] \quad (1)$$

with h and w respectively the metallic stripe thickness and width (see Supplementary Fig. 5a). Note that the magnetic sample thickness $t = 3$ nm differs from $h = 16$ nm, which includes non-magnetic layers. A skyrmion at the center of the stripe will tend to minimize its energy by shifting towards the edge where the field is parallel to its core magnetization orientation, thus in a direction parallel to $pj\mathbf{y}$.

Under the same current, the skyrmion moves along the current direction and therefore feels a gyrotropic force. For the initial motion, i.e. purely along $\mathbf{x}$, this force is along $-pv_x\mathbf{y} \propto -pj\mathbf{y}$.

It is remarkable to note that these two forces have the same dependence on both j and p and are always antiparallel (see Supplementary Fig. 5b). Note that this property is valid only in our specific case where skyrmions move along the current flow direction. Therefore, as our experiments indicate a deflection which is coherent with the direction predicted by the gyrotropic effect, there is no ambiguity on the dominant mechanism.

At the center of the stripe, the corresponding forces can be quantitatively evaluated. In a large region in the stripe center, the Oersted field varies linearly with y as

$$B_z \cong \frac{\mu_0 jh}{4\pi}\frac{8y}{w}. \quad (2)$$

Deriving the energy of a skyrmion of radius R with respect to y gives the Oersted field force

$$\mathbf{F}_{\mathrm{Oe}} = 2M_{\mathrm{s}}\frac{\partial B_z}{\partial y}\pi R^2 t\mathbf{y} = 4\mu_0 M_s j\frac{hR^2 t}{w}\mathbf{y}. \quad (3)$$

The gyrotropic force is

$$\mathbf{F}_{\mathrm{G}} = -\frac{4\pi\mu_0 M_{\mathrm{s}} t}{\gamma_0} v_x \mathbf{y} \quad (4)$$

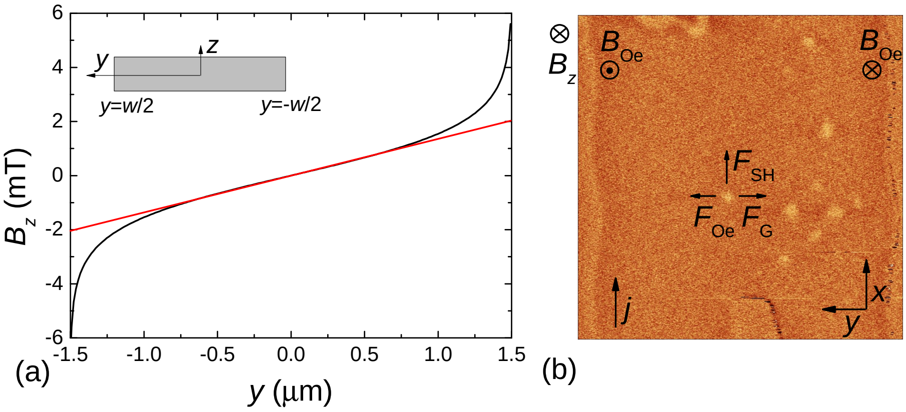

Supplementary Figure 5 | Effect of the Oersted field on the skyrmion deflection. (**a**) Variation of the z component of the Oersted field along the transverse direction to the stripe for $j = 3 \times 10^{11}$ A m^{-2}. Inset shows sketch of the stripe, aligned along x, for the Oersted field calculation. (**b**) Superposed on the experimental result from Fig. 3b, forces acting on a skyrmion with a positive core polarisation $p = 1$. The current j along x pushes the skyrmion along the stripe through the spin Hall effect (related force: $F_{\rm SH}$) which acquires a velocity v in the same direction. The current also produces an Oersted field (positive at the left stripe edge) which attracts the skyrmion to the left edge (related force: $F_{\rm Oe}$). Due to the velocity and its particular topology, the skyrmion experiences a gyrotropic force, which attracts the skyrmion toward the right edge (related force: $F_{\rm G}$). The experimental deflection toward the right edge proves the dominant role of the gyrotropic force with respect to the Oersted field-induced deflection.

and the force exerted by the spin Hall effect is

$$\mathbf{F}_{\rm SH} \approx \frac{\hbar}{2e}\theta_{\rm SH} j \pi^2 R \mathbf{x} \tag{5}$$

which is valid for a large skyrmion diameter as compared to the DW width.

Considering the parameters of our experiment ($M_{\rm s} = 850$ kA m^{-1}, $t = 3$ nm, $\theta_{\rm SH} \approx 0.1$[8], $h = 16$ nm and $w = 1$ or 3 μm) we can estimate these forces. At our maximum current density $j = 4.4 \times 10^{11}$ A m^{-2}, the skyrmion velocity is about $v_x \approx 60$ m s^{-1}, which gives values summarized in Supplementary Table 1 where the two estimations of $F_{\rm Oe}$ correspond to the two experimental setups shown in Figs. 2 and 3 respectively.

R	60 nm	80 nm	100 nm	150 nm
$\lvert F_{\rm SH}\rvert$	8.6	11.0	14.0	21.4
$\lvert F_{\rm G}\rvert$	11.0	11.0	11.0	11.0
$\lvert F_{\rm Oe}\rvert$	0.3	0.6	0.9	2.0

(a)

R	60 nm	80 nm	100 nm	150 nm
$\lvert F_{\rm SH}\rvert$	8.6	11.0	14.0	21.4
$\lvert F_{\rm G}\rvert$	11.0	11.0	11.0	11.0
$\lvert F_{\rm Oe}\rvert$	0.1	0.2	0.3	0.7

(b)

Supplementary Table 1: Forces acting on a moving skyrmion. Forces acting on a moving skyrmion of a radius R in (a) 1 μm and (b) 3 μm wide wires. Values of spin Hall force $F_{\rm SH}$, gyrotropic force $F_{\rm G}$ and Oersted field force $F_{\rm Oe}$ are given in pN.

This quantitative estimation proves again that the gyrotropic deflection dominates over the Oersted field-induced deflection.

Supplementary Note 4. SKYRMION MOTION UNDER SPIN HALL EFFECT IN A PERFECT MEDIUM

In general, two very different skyrmions motion regimes are known[9,10]. For a free-standing skyrmion[9], the solution of the Thiele equation gives a velocity having both longitudinal and transverse components

$$v_x = \frac{\alpha \mathcal{D}_{xx} F_{\rm SH} - G F_{\rm Oe}}{(\alpha \mathcal{D}_{xx})^2 + G^2}, \tag{6}$$

$$v_y = \frac{\alpha \mathcal{D}_{xx} F_{\rm Oe} + G F_{\rm SH}}{(\alpha \mathcal{D}_{xx})^2 + G^2}. \tag{7}$$

with α the Gilbert damping, $\mathcal{D}_{xx}$ the diagonal element of the dissipative tensor and G the z component of the gyrovector. We note that one has to take into account the absolute sign of the acting forces in these equations.

The second mechanism has been proposed by Sampaio *et al.*[10]. In a confined geometry where the lateral forces are balanced by edge repulsion, the skyrmion velocity increases significantly to

$$v_x = \frac{F_{\mathrm{SH}}}{\alpha \mathcal{D}_{xx}}. \tag{8}$$

To quantitatively express the forces acting on the moving skyrmion, we need to estimate G and $\mathcal{D}_{xx}$. On the one hand, from the interpretation of the gyrovector as the surface covered by the magnetic texture on the unit sphere[11], we know that the gyrovector value is, independently of the skyrmion profile,

$$G = -4\pi \frac{\mu_0 M_{\mathrm{s}} t}{\gamma_0}. \tag{9}$$

On the other hand, the value of the dissipation depends on the skyrmion profile. It is expressed as

$$\mathcal{D}_{xx} = \frac{\mu_0 M_{\mathrm{s}} t}{\gamma_0} \int \left(\frac{\partial \mathbf{m}}{\partial x}\right)^2 \mathrm{d}x\mathrm{d}y = \frac{\pi \mu_0 M_{\mathrm{s}} t}{\gamma_0} \int \left[\left(\frac{d\theta}{\mathrm{d}r}\right)^2 + \frac{\sin^2\theta}{r^2}\right] r\mathrm{d}r, \tag{10}$$

the latter equality applying to a profile with revolution symmetry.

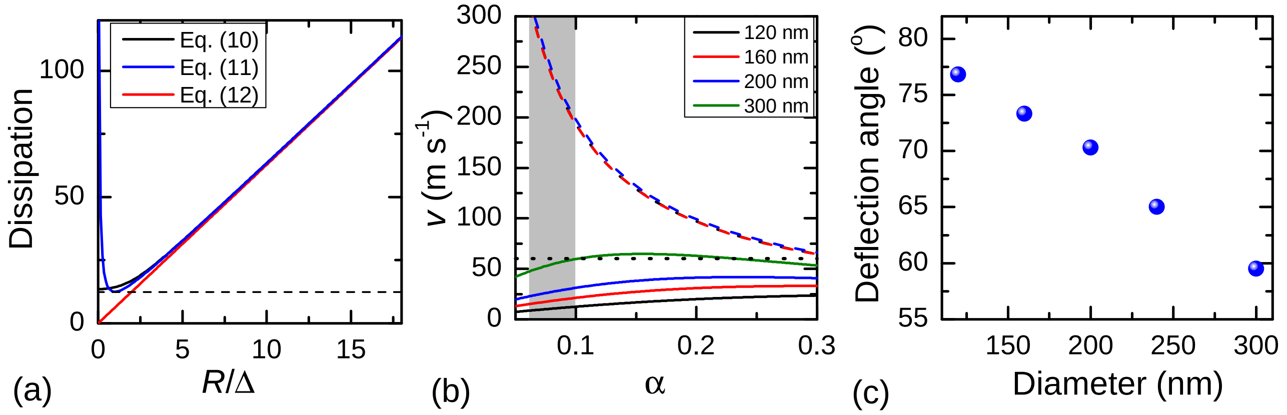

Supplementary Figure 6 | Skyrmion dynamics. (**a**) Calculated diagonal element of the dissipative tensor using Supplementary Eqs. (10), (11) and (12) normalized by the prefactor $\frac{\mu_0 M_{\mathrm{s}} t}{\gamma_0}$. The Belavin-Polyakov limit[14] (4π) is plotted in dashed line. (**b**) Calculated skyrmion velocities using Supplementary Eqs. (6) (lines) and (8) (dashed) in case of $w = 1$ μm, θ_{SH}=0.1 and $j = 0.44$ TA m^{-2} for skyrmions of various diameters. The dotted line corresponds to the experimental value. (**c**) Deflection angle at $j = 0.44$ TA m^{-2} and $\alpha = 0.08$ for various skyrmion sizes.

The numerical evaluation of $\frac{\gamma_0}{\mu_0 M_{\mathrm{s}} t}\mathcal{D}_{xx}$ using the planar 360° Bloch DW profile, that was shown to fit well the skyrmion radial profiles as observed by spin-polarized STM[12], is shown in Supplementary Fig. 6a, as a function of the reduced ratio R/Δ. This result is compared to two approximations of the radial integral in Supplementary Eq. (10), relevant when one has $R \gg \Delta$. In such a case one can put $r = R$ when integrating over the DW profile, and assume that $\theta(r)$ has the Bloch wall profile. The solution leads to

$$\mathcal{D}_{xx} \approx \frac{\mu_0 M_{\mathrm{s}} t}{\gamma_0} 2\pi \left(\frac{R}{\Delta} + \frac{\Delta}{R}\right), \tag{11}$$

when one considers both terms, whereas with only the first term one gets

$$\mathcal{D}_{xx} \approx \frac{\mu_0 M_{\mathrm{s}} t}{\gamma_0} 2\pi \frac{R}{\Delta}. \tag{12}$$

One sees that for $R > \Delta$ the approximation of Supplementary Eq. (11) corresponds well to the result for the 360° DW profile. The dissipation values are distinctly smaller than those quoted in Ref. 13, in which a very schematic DW profile was used to compute the first term of radial integral of Supplementary Eq. (10). Note that, even if the

dissipation depends on the skyrmion profile, it cannot take any value. Indeed, Supplementary Eq. (10) shows that the trace of the dissipation tensor ($\mathcal{D}_{xx} + \mathcal{D}_{yy}$) is proportional to the exchange energy. At this point, it is useful to recall that Belavin and Polyakov have proved mathematically[14] that the exchange energy is bounded by the absolute value of the skyrmion number, the equality being obtained for the Belavin-Polyakov profile. This means, in the present notations, that

$$\mathcal{D}_{xx} + \mathcal{D}_{yy} > 2|G|. \tag{13}$$

Thus for a profile with revolution symmetry where $\mathcal{D}_{xx} = \mathcal{D}_{yy}$, and a skyrmion number ± 1 one has, mathematically,

$$\mathcal{D}_{xx} > 4\pi \frac{\mu_0 M_s t}{\gamma_0}. \tag{14}$$

After this digression on the evaluation of the dissipation tensor of a skyrmion, we come back to numerical estimates. With $R = 80$ nm and $\Delta = 12.9$ nm (see Fig. 1b of the main text; note that this value is very different from the 1-layer calculation $\sqrt{A/K_{\text{eff}}}$ due to dipolar effects), we obtain $\mathcal{D}_{xx}\gamma_0/\mu_0 M_s t \approx 40$. To express the effect of α on the velocity, the calculated velocities are plotted in Supplementary Fig. 6b.

In the case of the first regime the velocity at $j = 4.4 \times 10^{11}$ A m^{-2} along x is expected to be 18 m s^{-1} resulting in a deflection angle of $\approx 73°$ (see Supplementary Fig. 6c), while for the second regime, a velocity of 240 m s^{-1} is expected (Note that, in this case, the velocity strongly depends on α, for which our rough estimation implies a large uncertainty on the expected velocity.). The experimental value (60 m s^{-1}) is close to the free-standing skyrmion solution. The fact that the calculated skyrmion velocities in the confined skyrmion regime are independent of the skyrmion size arises from the proportionality of F_{SH} and $\mathcal{D}_{xx}$ to R for $R > \Delta$.

Supplementary References

[1] Di, K. *et al.* Direct observation of the Dzyaloshinskii-Moriya interaction in a Pt/Co/Ni film. *Phys. Rev. Lett.* **114**, 047201 (2015).

[2] Belmeguenai, M. *et al.* Interfacial Dzyaloshinskii-Moriya interaction in perpendicularly magnetized Pt/Co/Alox ultrathin films measured by Brillouin light spectroscopy. *Phys. Rev. B* **91**, 180405 (2015).

[3] Grolier, V. *et al.* Unambiguous evidence of oscillatory magnetic coupling between Co layers in ultrahigh vacuum grown Co/Au (111)/Co trilayers. *Phys. Rev. Lett.* **71**, 3023 (1993).

[4] Vansteenkiste, A. *et al.* The design and verification of MuMax3. *AIP Advances* **4**, 107133 (2014).

[5] Bauer, M. *et al.* Deroughening of domain wall pairs by dipolar repulsion. *Phys. Rev. Lett.* **94**, 207211 (2005).

[6] Kooy, C. & Enz, U. Experimental and theoretical study of the domain configuration in thin layers of BaFe12O19. *Philips Res. Rep.* **15**, 7 (1960).

[7] Rohart, S. & Thiaville, A. Skyrmion confinement in ultrathin film nanostructures in the presence of Dzyaloshinskii-Moriya interaction. *Phys. Rev. B* **88**, 184422 (2013).

[8] Nguyen, M.-H., Ralph, D. & Buhrman, R. Spin torque study of the spin Hall conductivity and spin diffusion length in platinum thin films with varying resistivity. *Phys. Rev. Lett.* **116**, 126601 (2016).

[9] Iwasaki, J., Mochizuki, M. & Nagaosa, N. Universal current-velocity relation of skyrmion motion in chiral magnets. *Nat. Commun.* **4**, 1463 (2013).

[10] Sampaio, J., Cros, V., Rohart, S., Thiaville, A. & Fert, A. Nucleation, stability and current-induced motion of isolated magnetic skyrmions in nanostructures. *Nat. Nanotech.* **8**, 839–844 (2013).

[11] Thiele, A. Steady-state motion of magnetic domains. *Phys. Rev. Lett.* **30**, 230 (1973).

[12] Romming, N., Kubetzka, A., Hanneken, C., von Bergmann, K. & Wiesendanger, R. Field-dependent size and shape of single magnetic skyrmions. *Phys. Rev. Lett.* **114**, 177203 (2015).

[13] Jiang, W. *et al.* Direct observation of the skyrmion Hall effect. *Nat. Phys.* **13**, 162–169 (2016).

[14] Belavin, A. & Polyakov, A. *Pis'ma Zh. Eksp. Teor. Fiz.* 22, 503 (1975). *JETP Lett.* **22**, 245 (1975).
